# Cleaning and disinfection in health care settings during the COVID-19 outbreak

**Published:** 2020-09-01

**Authors:** Xiao Ying Liu, Yan Zhang, Hai Xia Tu, Astrid Leck

**Affiliations:** 1 RN, Staff Nurse: Orbis International, New York, USA.; 2 RN, Associate Director of Nursing: Wuhan Aier Eye Hospital, Wuhan city, Hubei Province, China.; 3 RN, Department of Infection Control & Quality Control Officer: Wuhan Aier Eye Hospital, Wuhan City, Hubei Province, China.; 4 Research Fellow and microbiologist: London School of Hygiene & Tropical Medicine, London, UK.

**Cleaning and disinfection of the health care environment plays an important role in reducing indirect transmission of SARS-CoV-2 – the virus responsible for COVID-19.**

**Figure:**
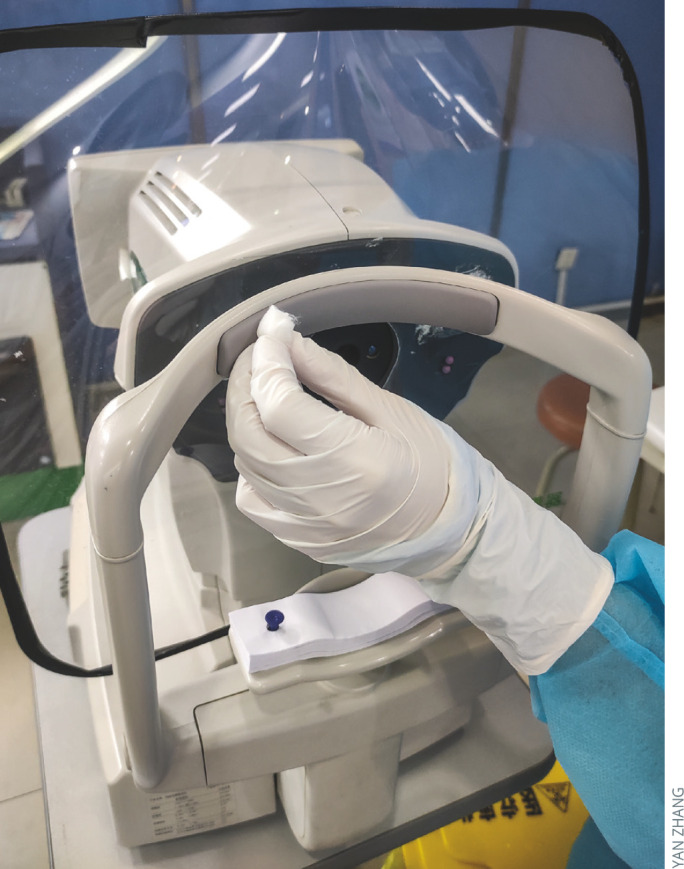
Thorough cleaning and disinfection of equipment and surfaces is essential. **CHINA**

SARS-CoV-2 can remain viable for between eight hours and several days, depending on the type of surface.^1^ Surfaces become contaminated when virus-containing droplets land on them, or when someone with contaminated hands touches these surfaces.

Decontamination of the health care environment is therefore vital. It includes cleaning, disinfection and the safe disposal of waste. In this article, we focus primarily on cleaning and disinfection; safe waste disposal will be discussed in more detail in a future issue of *Community Eye Health Journal*.

**Cleaning** is a process which removes contaminants, such as dust or microorganisms, and the body fluids (or organic matter) that shield them. **Disinfection** is the process by which any microorganisms that remain after cleaning are reduced to a level at which they are not harmful, which is only effective if the equipment or surface is thoroughly cleaned with a detergent solution beforehand.^2^

In the health care environment, we must clean and disinfect surfaces such as walls, floors, furniture, sinks and taps, stairway rails, touch screens, counter tops, door handles and light switches; and equipment such as torches, ophthalmoscopes, trial lenses, slit lamps, and wheelchairs, to name a few!

Standard cleaning and disinfection protocols continue to apply during the pandemic, but may have to take place more often. This article is based on guidance from the World Health Organization (**bit.ly/COV19clean**), national bodies and current research, but we also strongly recommend that you follow national guidelines.

## Management of cleaning and disinfection

It is important that cleaning and disinfection practices are closely monitored, and that personnel responsible for cleaning have the correct PPE and are trained properly.

In the hospital setting, the infection control team is responsible for developing and approving cleaning and disinfection policies and strategy. Assigning cleaning duties, setting up cleaning schedules and checklists, and monitoring cleaning and disinfection practices is the responsibility of matrons, domestic supervisors and service managers.

Appropriate PPE must be worn during preparation of cleaning products and while cleaning: heavy duty gloves, face mask, eye protection (safety goggles or a face shield), a gown and closed work shoes. Note: heavy-duty gloves must be cleaned and disinfected before moving from one area to another, e.g., when moving from the outpatients waiting room to an examination room, and vice versa.

Everyone who is responsible for cleaning in the health care environment must be trained in:

- Safe disinfectant preparation
- Cleaning methods and equipment use
- Standard precautions
- Risk assessment and transmission-based precautions.

## Preparation

Ideally, use fresh cloths and prepare fresh solutions of detergent and disinfectant for each cleaning shift. Rinse and dry mops and buckets between shifts. Detergents and disinfectant solutions applied from a bucket with a cloth or mop will become contaminated during cleaning and progressively less effective. Continued use of the same detergent or disinfectant solution may transfer microbes to each subsequent surface.

Always refer to the manufacturer’s instructions when preparing and using disinfectants. When selecting disinfectants for use on items of equipment, check the manufacturer’s guidance before you proceed.

Prepare the correct concentration of disinfectant and allow it to remain on the surface for long enough to achieve effective surface disinfection (see the manufacturer’s recommendations). Concentrations with inadequate dilution during preparation (too high or too low) may reduce their effectiveness. High concentrations increase the risk of exposing people to harmful chemicals and may also damage surfaces.

**Note:** After cleaning equipment or an area associated with a confirmed or suspected COVID-19 patient, discard solutions and cloths **immediately**.

Although there are some indications for non-touch disinfection methods like spraying and fumigation (fogging), these techniques are not recommended for routine disinfection of indoor spaces due to potential adverse health risks for the user and other people. Refer to Table 1 for indications. **Under no circumstances should a person be sprayed with disinfectant**.

## How to clean & disinfect: a guide

### Cleaning

- Clean surfaces thoroughly with a neutral detergent (soap and water). Begin with the cleanest areas first, then move to the more contaminated areas. Clean surfaces that are touched less often before moving on to frequently touched surfaces.
- Take care to clean all surfaces, even if they are not visibly dirty. Scrubbing may be necessary to first remove and reduce visible dirt, debris and other organic matter (e.g., blood, secretions and excretions). Organic matter, or ‘soil’, can prevent direct contact of a disinfectant with a surface, so that the disinfectant can’t reach or destroy the microorganisms that may be present.

Clean and disinfect surfaces more often in areas with high traffic, such as outpatient areas and rooms where staff members don and doff PPE.^3^

Use clean or disposable cloths or paper towels to apply a chemical disinfectant (a chlorine-releasing agent, or 70% ethanol or isopropanol) after cleaning to destroy any remaining microorganisms. Dispose of waste carefully, following standard procedures.

### How often?

During the COVID-19 pandemic the frequency with which all routine cleaning and disinfection takes place should be increased. Give priority to frequently touched surfaces or contact points such as door handles, for example.^4^

However, it is also important to assess the risk as low, moderate, or high – based not only on the room or area but also on what patient care activities or procedures take place in that space. For example, patient waiting areas are low-risk areas (provided patients are spaced 1–2 metres apart, and there is adequate ventilation), but operating theatres are high-risk areas.

**Selection of disinfectants**

Like other coronaviruses, SARS-CoV-2 is very susceptible to disinfectants. Refer to Table 1 for examples of widely used and available disinfectants. Each one has its advantages and side effects; both the scope of application and chemical characteristics of a disinfectant should be considered, alongside local guidelines, before choosing one.

**Table 1:** Disinfectants commonly used against the novel coronavirus in health facilities

Disinfectant	Concentration	Application scope
Chlorine-releasing disinfectant products) containing sodium chlorite, sodium hypochlorite or chlorine dioxide)	0.5% (5,000 ppm) 0.1% (1,000 ppm)	Faecal, bodily fluid or blood, vomit from infected patients, large spills Contaminated object surfaces, floors, walls, equipment surfaces*
Alcohols	70%	Object surfaces, medical equipment surfaces,*ophthalmic equipment*
Hydrogen peroxide (non-chlorine bleach)	≥ 0.5%	Fogging vapour for terminal cleaning, periodic deep cleans, or outbreak cleans of a ward environment; for enhanced cleaning

^*^ When a chlorine-containing disinfectant or alcohol is used to disinfect the surface of medical equipment, it is important to consult your medical technicians or manufacturers. Some equipment, in particular metal equipment and electronics, may be sensitive to certain chemicals and they may cause damage. When non-alcoholic disinfectants are applied to surfaces or equipment, wipe down with alcohol or distilled water to remove residues. To prevent electrical shock or damaging electronics, some frequently touched surfaces (such as light switches, phones, computer and keyboards) may be disinfected using 70% alcohol.

**Table 2:** Guidelines for cleaning and disinfection of health care surfaces and equipment

Healthcare area	Surfaces (examples)	Frequency
**Reception or out-patient waiting area**	Desk phones, counter tops, keyboards, touch screens	At least twice daily with 70% ethanol/isopropanol or products specified by the manufacturer Frequently touched surfaces to be disinfected after each patient visit
	Lifts, handrails, door/toilet handles, light switches	As frequently as possible – at least twice daily – and whenever visibly soiled or known to be contaminated with secretions, excretions or body fluids
	Hallways, floors, (walls), furniture	At least twice daily, whenever visibly soiled and when known to be contaminated with secretions, excretions or body fluids. Focus on areas that are touched less frequently, then on frequently touched surfaces, then floors (last)
	Toilets and washrooms	At least three times per day Frequently touched surfaces, including door handles, light switches, counters, taps, then sink bowls, then toilets and finally floor (in that order) Avoid sharing toilets between staff and patients
**Screening or triage**	Counters, tables, pens, clipboards, thermometer	At least twice daily Clean and disinfect frequently touched surfaces and surfaces that may have been exposed to respiratory droplets between each patient care episode Clean and disinfect equipment after each use
**Clinic room**	Slit lamp chin rest, table, chair, ophthalmic equipment	Clean and disinfect frequently touched surfaces and surfaces that may have been exposed to respiratory droplets between each patient care episode Clean and disinfect equipment after each use
**Inpatient rooms, wards**	Beds, chairs, floors	At least twice a day, preferably three times a day Focus on frequently touched surfaces, starting with shared or common surfaces, then move to each patient bed. Use a new cloth for each bed if possible. Clean floors last.
**Operating theatre**	As per hospital policy	As per hospital policy
